# Management of Urgent Bleeding in Patients with Hemophilia A: Focus on the Use of Emicizumab

**DOI:** 10.1055/s-0044-1785525

**Published:** 2024-04-17

**Authors:** Víctor Jiménez-Yuste, María T. Álvarez-Román, Rubén Berrueco, Santiago Bonanad, José M. Calvo-Villas, Rebeca González-González, José R. González Porras, Ramiro J. Núñez-Vázquez, Manuel Rodríguez-López

**Affiliations:** 1 Hematology Department, La Paz University Hospital-IdiPaz, Universidad Autónoma, Madrid, Spain; 2 Pediatric Hematology Unit, Hospital Sant Joan de Deu, Barcelona, Spain; 3 Hematology Department, Hospital Universitari i Politècnic La Fe, Valencia, Spain; 4 Hematology Department, Hospital Universitario Miguel Servet, Zaragoza, Spain; 5 Emergency Department, Hospital Universitario Severo Ochoa, Leganés, Madrid, Spain; 6 Hematology Department, Hospital Clínico Universitario, Salamanca, Spain; 7 Hematology Department, Hospital Universitario Virgen del Rocío, Instituto deBiomedicina de Sevilla (IBIS), Sevilla, Spain; 8 Hematology Department, Hospital Álvaro Cunqueiro, Vigo, Spain

**Keywords:** hemophilia, emicizumab, emergency, bleeding, inhibitors

## Abstract

Management of patients with hemophilia A (HA) requires the knowledge and experience of specialized health care professionals. However, these patients may need to be attended in emergencies, outside the referral hospital, where health care professionals do not know about hemophilia and/or new innovative treatments.

This study aimed to develop a simple and practical algorithm that could be used in emergency situations by nonspecialized treaters in HA and bleeding with or without factor VIII (FVIII) inhibitors under emicizumab prophylaxis.

A group of experts agreed on a simple algorithm, easy to operate, adapted from previous international guidelines, and based on their clinical experience.

The proposed algorithm starts with identifying the patient, confirming the diagnosis of HA, prophylaxis with emicizumab, and/or use of other treatments. After stabilizing the patient and stratifying the bleeding risk, the patient is managed according to the presence/absence of FVIII inhibitors. Patients without FVIII inhibitors should receive FVIII concentrate. Dose and follow-up depend on bleeding localization and severity. Patients with FVIII inhibitors should preferably receive recombinant activated factor VII as bypass agent. A basic coagulation assay, FVIII assessment, and FVIII inhibitors detection assays are necessary in an emergency. However, these tests should be interpreted with caution and appropriately chosen, as emicizumab may alter the results.

The management of patients with HA is challenging in emergency situations, especially if they are treated with new agents. Nonspecialized in coagulopathies health care professionals have limited understanding of the disease, highlighting the need for an algorithm to assist them in making informed decisions.

## Introduction

Hemophilia A (HA), which is caused by congenital factor VIII (FVIII) deficiency, is a well-known coagulation disorder despite its rarity. It affects 1 in 10,000 cases in the population. 1 Due to its low prevalence, expertise is scarce outside the hemophilia care centers. Since their first edition, the World Federation of Hemophilia guidelines have promoted the creation of hemophilia treatment centers that, through multidisciplinary teams, would be able to bring knowledge, experience, and a comprehensive approach to patients with hemophilia. 2

The classic treatment of hemophilia has been based on the use of clotting factor concentrates, first from donor plasma and subsequently produced by genetic engineering to give rise to recombinant factors with a longer half-life, which in the case of HA is FVIII. 2 3 4 However, the development of inhibitory antibodies against FVIII is relatively common. 5 6 Up to 20 to 40% of patients with HA develop inhibitors against FVIII. 2 These inhibitors decrease the efficacy of conventional treatments with factor concentrates, increasing the rate of bleeding episodes and negatively impacting patients' quality of life and life expectancy. To address this situation, different agents are utilized, including bypass agents like activated prothrombin complex concentrates (aPCCs) and recombinant activated factor VII (rFVIIa) or more recently FVIII-mimetic bispecific antibodies (emicizumab). 2 3 4 7

Emicizumab is a chimeric bispecific monoclonal antibody that binds to FIXa and FX, mimicking the cofactor function of FVIII in patients with HA. This drug allows the activation of FX without the need for FVIII intervention, thus initiating the final common pathway that leads to thrombin generation. 8 9 The main benefits of emicizumab are its subcutaneous route of administration; prolonged half-life, which allows maintenance dosing up to every 4 weeks; high efficacy in the prevention of bleeding in HA patients with or without FVIII inhibitors. Data from studies have shown that long-term use of emicizumab prophylaxis resulted in statistically significant and clinically meaningful reductions in bleeding rates and target joint resolution in patients with HA with a favorable safety profile, regardless of age, FVIII inhibitor status, or dosing regimen. 8 10 11 12 13 14 These findings show that emicizumab is recommended for patients of all ages with HA who have FVIII inhibitors for regular prophylaxis of bleeding episodes. It is also recommended for those with severe (FVIII <1%) and moderate (FVIII ≥1 and ≤5%) HA without FVIII inhibitors. 8 Nevertheless, as emicizumab does not normalize completely the coagulation process, full protection from all bleeding episodes is not expected. 15

Under normal conditions, patients with HA go to their referral center for treatment. However, there may be situations in which they come at a time when a specialized hematologist is not available, does not have a referral center nearby, or must be treated at an emergency center with health care personnel without experience with hemophilia treatment. This is even more complicated due to the arrival of new innovative drugs such as emicizumab. This treatment has a different mechanism of action, its management differs from the other treatments used in hemophilia, and modifies the results of basic laboratory tests. Although there are international protocols and guidelines widely used by specialized health care professionals, 16 17 18 19 20 they do not solve the problem of managing these patients in emergency situations in which specialized personnel are not always available.

Therefore, with the aim of adapting these guidelines and recommendations to the reality of patients with HA with or without FVIII inhibitors under prophylaxis with emicizumab in emergency situations, after a review of the most recent literature on the topic and several discussion meetings, a group of experts in hemophilia agreed on a simple and practical algorithm adapted from previous international guidelines and based on their clinical experience that can be used in emergency situations by nonspecialized personnel.

## Basic Recommendations for the Treatment of a Patient with Hemophilia A in the Event of a Bleeding Emergency

Patients with hemophilia, apart from being trained in self-management, are instructed to recognize bleeding and its management at home, especially joint and muscle bleeding. In addition, it is also important that family members and caregivers recognize the subtle signs of bleeding in young children with hemophilia.

In general, the main treatment for bleeding is with concentrates of FVIII as prescribed by health care professionals. Local measures should be considered in the case of mild bleeding, that is, compression, ice, and topical hemostatic agents, including tranexamic acid; moreover, systemic administration of tranexamic acid should be added. In people with HA and FVIII inhibitors, it would be necessary to treat bleeding with a bypass agent according to the hematologist's indications. Despite these basic recommendations, patients often need to be treated in an emergency department.

## General Recommendations for Management of a Patient with Hemophilia in Emergency Situations

If someone with hemophilia seeks medical attention in an emergency situation, it is important that they are evaluated promptly. Even seemingly minor complications can worsen if they are made to wait for too long. 2 These patients require stabilization or treatment similar to other subjects in an emergency situation. 21 However, there are some precautions to be taken into account. For instance, it is recommended that intravenous access be performed by experienced personnel, as different attempts may result in hematoma formation. Unless it is necessary to infuse large volumes of fluid or blood, it is preferable to opt for small caliber catheters. 21

Emicizumab is a drug indicated for the prophylactic treatment of HA that is not useful for treating acute bleeds. Patients receiving emicizumab who experience breakthrough bleeding should be treated based on whether they have FVIII inhibitors or not. In the absence of inhibitor, they are treated with FVIII concentrates at doses considered necessary to achieve hemostasis. In the presence of inhibitor, a bypass agent should preferably be used (see details in the next section of the algorithm). 16 17 18 19 21

## Algorithm for the Management of Patients with Hemophilia A in Emergency Situations

The algorithm starts when a patient arrives to the emergency department with a bleeding, a potential bleeding problem, or requiring an invasive procedure. According to the recommendations of the emergency departments, the first step is to identify and stabilize the patient in case of acute bleeding. It is recommended, in the first instance, to request the laboratory tests that will guide future decisions: a complete blood count, a basic coagulation study, and the measurements of FVIII levels and its inhibitor from each center, based on their respective capabilities.

### Phase I: Patient Identification

The identification of the patient is the first step of the proposed algorithm and a key step because it allows guiding the health professional(s) in one direction or another, saving time in urgent situations. If patients are outside their referral hospital and electronic medical records are not available, patients are advised to carry, for example, an identification card or a small report. The experts' recommendation about the minimum data that the report should contain are the diagnosis of HA, severity (mild, moderate, or severe), the presence or absence of FVIII inhibitors, whether the patient is on treatment with emicizumab, what treatment has been prescribed in case of acute bleeding (FVIII or rFVIIa), and the contact details of the referral center. In those with FVIII inhibitors, the type of anamnestic response (high/low responder) and the last available inhibitor titer should be cited, although the implications rely on the treatment prescribed for acute bleeding. This information would be very useful in emergency situations so that the patient can be attended as quickly as possible.

**Fig. 1:**
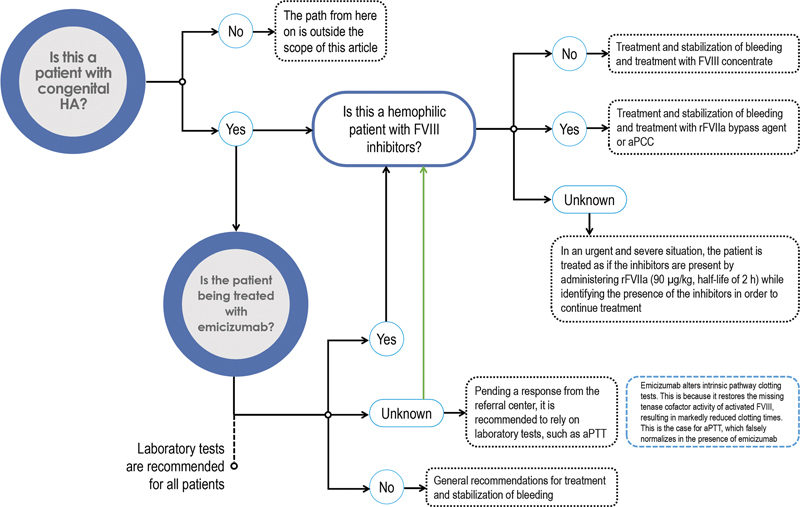
Identification of patients with hemophilia A. aPCC, activated prothrombin complex concentrate; aPTT, activated partial thromboplastin time; FVIII, coagulation factor VIII; HA, hemophilia A; rFVIIa, recombinant activated factor VII.

Nevertheless, for the correct identification of the patient, it is recommended to contact the patient's referral center, but in case this is not possible, or there is not enough time, it is suggested to follow the algorithm shown in Fig. 1 . The present document only addresses the situation in which the patient has congenital HA.

After verifying that the patient has HA, it is necessary to know if the patient is being treated with emicizumab. If yes, check whether the patient has FVIII inhibitors. If treatment for hemophilia is unknown, while obtaining a response from the referral center, it is recommended to rely on laboratory tests, such as activated partial thromboplastin time (aPTT). It is important to note that false normalization of aPTT may be obtained in patients treated with emicizumab.

Depending on whether the patient has FVIII inhibitors or not, different recommendations will be followed according to the treatment algorithm for bleeding in HA (see “Phase III: Treatment for Bleeding” section for details). If the presence of FVIII inhibitors is unknown, either because the patient is unconscious or in an urgent and severe situation, the patient is treated as if the inhibitors are present by administering rFVIIa (with a half-life of 2 h) while identifying the presence of the inhibitors in order to continue treatment.

### Phase II: Stabilization of the Patient

Patients with hemophilia may present to the emergency room for any cause unrelated to their disease. Therefore, it is always recommended to perform all appropriate instrumental (i.e., ultrasonography) and laboratory evaluations to confirm or reasonably exclude the presence of bleeding and to assess its severity. In addition to the location or type of bleeding, it is important to consider the hemodynamic status of the bleeding patient. Recommendations for different kind of bleedings, polytrauma, or potential bleeding are described below.

**Fig. 2:**
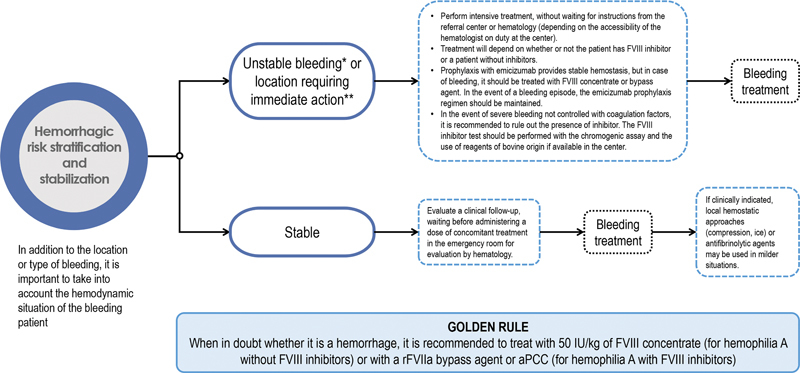
Stabilization and treatment of the patient. *Hemodynamic instability defined by shock index (heart rate/systolic blood pressure) >1. **Locations requiring rapid action: head and neck bleeding, compartment syndrome, central nervous system, gastrointestinal, major surgery, and major trauma, among others. aPCC, activated prothrombin complex concentrate; FVIII, coagulation factor VIII; rFVIIa, recombinant activated factor VII.

If the bleeding is mild and the patient is stable, clinical follow-up should be assessed, waiting for evaluation by the hematologist before administering a concomitant dose of treatment in the emergency room. If clinically indicated, local hemostatic approaches (e.g., compression, ice) or antifibrinolytic agents may be used in milder situations ( Fig. 2 ). Treatment of bleeding is described in section “Phase III: Treatment for Bleeding.”

If the bleeding is severe or critical, with unstable bleeding (defined by shock index [heart rate/systolic blood pressure] >1) or in a location that requires immediate treatment (head and neck, compartment syndrome, central nervous system, gastrointestinal, major surgery and major trauma, among others), intensive treatment will be performed without waiting for instructions from the referral center or Hematology department ( Fig. 2 ; see section “Phase III: Treatment for Bleeding” for details).

It is important to keep in mind the golden rule: when in doubt whether it is a hemorrhage, for patients with HA without FVIII inhibitors it is recommended to treat with 50 IU/kg of FVIII concentrate. For patients with HA with FVIII inhibitors, an rFVIIa bypass agent should be preferred (given the concomitant use of emicizumab in large proportion of cases) or aPCC. This rule should be applied, above all, in situations that do not admit of delay (severe and/or life-threatening bleeding, before waiting for possible complementary tests).

### Phase III: Treatment for Bleeding

**Fig. 3:**
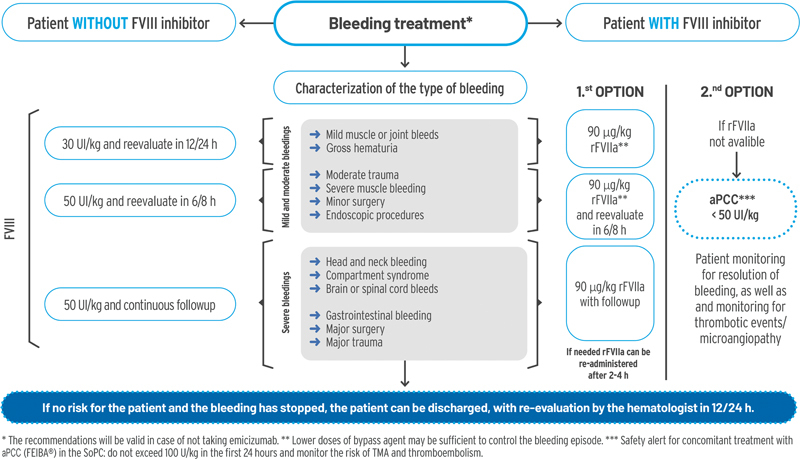
Bleeding treatment of patients with hemophilia A. aPCC, activated prothrombin complex concentrate; FVIII, coagulation factor VIII; rFVIIa, recombinant activated factor VII; TMA, thrombotic microangiopathy.

Once the patient has been stabilized (has stopped bleeding), treatment is performed according to the bleeding risk (mild, moderate, or severe) and the presence/absence of FVIII inhibitors ( Fig. 3 ). The following recommendations are valid both for patients taking emicizumab and those not taking emicizumab.

#### Patients without Factor VIII Inhibitors

Although prophylactic treatment with emicizumab provides stable hemostasis, in the event of bleeding, and/or some procedures such as major surgeries, FVIII replacement therapy should be used at a dose appropriate to the bleeding episode. In any case, in a bleeding event, the emicizumab prophylaxis regimen should be maintained.

In the case of mild muscle or joint bleeding or gross hematuria, the use of 30 IU/kg of FVIII concentrate is recommended, with a reevaluation at 12/24 hours. 17 To avoid a renal colic related to blood clots in the ureters in patients with gross hematuria, before the administration of FVIII concentrate it is recommended to give intravenous fluids until the color of the urine changes to a pinker shade. 2 22

If there has been moderate trauma, severe muscle bleeding, minor surgery, or an endoscopic procedure has been performed, 50 IU/kg of FVIII concentrate is recommended. Dose reduction of FVIII concentrate is conceivable for minor surgical procedures but not in children, who often show less favorable pharmacokinetics. Dental extractions and skin or other biopsies where adjuvant local hemostasis is possible would be excluded.

Finally, in the case of severe hemorrhages, such as those caused by bleeding in the head and neck, compartment syndrome, central nervous system, gastrointestinal, major surgery and major trauma, it is recommended to administer 50 IU/kg of FVIII concentrate with continuous monitoring of the patient. 17

If there is no risk to the patient and the bleeding has stopped, the patient may be discharged, but should be reevaluated by the Hematology Service in 12 to 24 hours.

#### Patient with Factor VIII Inhibitors

Prophylactic treatment with emicizumab provides stable hemostasis, but in case of bleeding, a bypass agent (rFVIIa) should be used. If rFVIIa is not available, aPCC could be used. If severe bleeding is not controlled with concentrates of clotting factors, it is recommended to rule out the presence of FVIII inhibitors. The FVIII inhibitors test should be performed with the chromogenic assay and the use of bovine reagents (to determine endogenous FVIII levels) or with human reagents (to measure the activity of endogenous FVIII and emicizumab; see section “Factor VIII Assessment”).

The use of rFVIIa is the preferred option. Depending on the bleeding episode, it would be used intensively at a dose of 90 μg/kg, monitoring the patient according to the severity of bleeding: every 6/8 hours for mild/moderate bleeding or continuously for severe bleeding. According to expert opinion, lower doses of bypass agent may be sufficient to control the bleeding episode. If necessary, rFVIIa administration can be repeated after 2 to 4 hours.

If rFVIIa is not available or an efficient response to this bypass agent is not achieved, aPCC would be used at a dose <50 IU/kg (not exceeding 100 IU/kg in the first 24 h), monitoring the risk of thrombotic microangiopathy (TMA) and thromboembolism until resolution of bleeding. It is important to note that serious and potentially life-threatening adverse effects have been observed using concomitant aPCC and emicizumab, such as TMA and thromboembolism, so both events should be closely monitored. 11

In patients with FVIII inhibitor at low titer, in the case of severe bleeding or failure of rFVIIa, the possible use of FVIII concentrates should be considered, although such indication and laboratory monitoring are up to specialized centers.

In both situations, if there is no risk to the patient and the bleeding has stopped (mild or moderate), the patient can be discharged, with reevaluation at 12 to 24 hours by the Hematology Service.

## Laboratory Tests and Precautions to be Considered if Emicizumab is Used

**Fig. 4:**
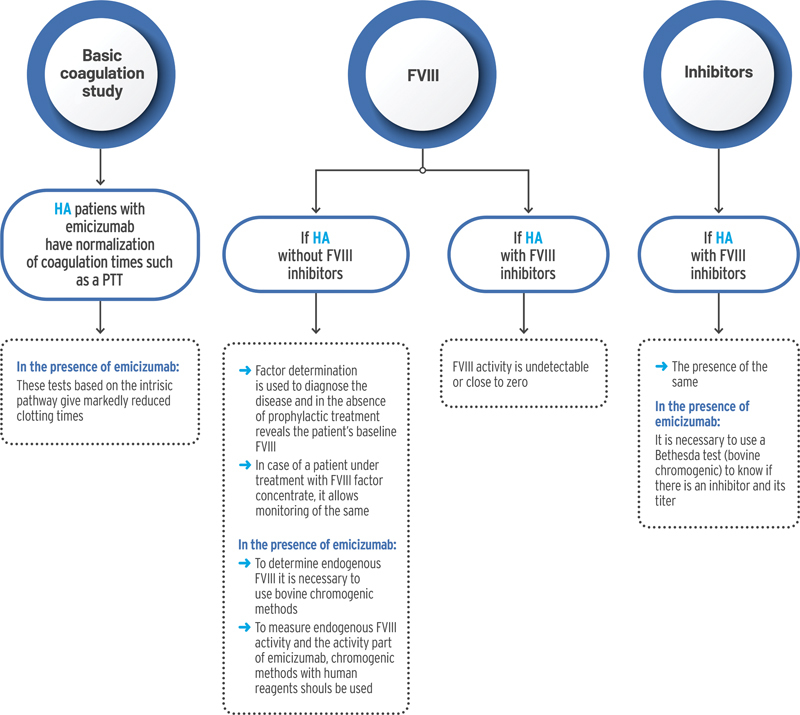
Laboratory tests and precautions to be taken into account if emicizumab is used. FVIII, coagulation factor VIII; HA, hemophilia A; PTT, partial thromboplastin time.

The main laboratory tests that can be performed in patients with HA and acute hemorrhage are the basic coagulation test, a complete blood count including platelet count, FVIII assessment, FVIII inhibitors detection, and other global hemostasis techniques (thrombin generation test and viscoelastometric techniques such as thromboelastography and rotational thromboelastometry). The first two tests are necessary in an emergency. 16 17 18 19 In practice, baseline FVIII determination is not performed always in a well-identified patient. On the contrary, the subsequent follow-up if the patient has to receive treatment should include FVIII measurement. On the other hand, quantifying an inhibitor in an emergency situation may be ambitious, although this should be aimed at, especially, in patients with unknown inhibitor “status.” Fig. 4 shows laboratory tests and precautions to be taken into account if emicizumab is used.

### Basic Coagulation Assay

The basic coagulation test would include prothrombin time test, partial thromboplastin time, and fibrinogen test (preferably fibrinogen Clauss assay if derived fibrinogen <150 mg/dL). 8 23 In patients with HA treated with emicizumab it is important to consider several aspects. On one hand, patients with HA have prolonged clotting times. On the other hand, emicizumab alters intrinsic pathway clotting tests. This is because it restores the missing tenase cofactor activity of activated FVIII, resulting in markedly reduced clotting times. This is the case for aPTT, which falsely normalizes in the presence of emicizumab. 24 25 26

### Factor VIII Assessment

For FVIII assessment the recommendation is to use, if available, both the one-stage coagulometric method and the chromogenic method with bovine reagents. 27 However, it is important to differentiate the assessment of FVIII in patients with or without FVIII inhibitors.

If FVIII inhibitors are not present, the assessment of FVIII might be useful to diagnose the disease. In the absence of prophylactic treatment it reveals the patient's baseline FVIII level. If the patient is receiving replacement therapy with FVIII concentrate, FVIII assessment allows monitoring of FVIII levels. However, in the presence of emicizumab, two considerations must be taken into account. On one hand, to determine endogenous FVIII levels it is necessary to use chromogenic methods with bovine reagents. On the other hand, to measure the activity of endogenous FVIII and emicizumab, chromogenic methods with human reagents must be used. In the case of patients with FVIII inhibitors, FVIII activity is undetectable or close to zero. 24 25 26

### Detection of Factor VIII Inhibitors

After exposure to FVIII concentrate, HA patients could develop alloantibody-type inhibitors. These inhibitors are usually uncorrected or partially corrected in the immediate mixing test; they are time- and temperature-dependent, and require incubation at 37°C for 2 hours. To confirm their presence and quantify them, the Bethesda test with the Nijmegen modification should be performed. 28 In the presence of emicizumab, it is necessary to use the Bethesda test (chromogenic method with bovine reagents) to know if there is an inhibitor and its titer. 24 25 26

## Conclusion

The management of patients with HA is a challenge in emergency situations. The lack of knowledge about the management of the disease by nonspecialized health care personnel makes it necessary to develop an algorithm to help them make the most appropriate decisions, considering factors such as the presence or absence of FVIII inhibitors, or treatment with emicizumab, which can affect the results of many of the coagulation tests routinely performed on these patients.

In addition to the algorithm, and to facilitate patient identification in situations requiring immediate assistance, it would be very useful for patients with HA to have an identification card, either physical or digital, on which all the patient's diagnostic and therapeutic information is recorded. In order to disseminate or implement the present algorithm in clinical practice, it would be very useful to present it at congresses and in emergency medicine journals, to obtain the support of the related scientific societies, to hold local meetings with the experts involved, and to create material in the form of posters or infographics.

Moreover, in order to optimize the whole process, coordination between the different levels of care and, within the hospital, between the different specialists involved in the management of these patients would be necessary.
